# Serum Long-Chain n-3 Polyunsaturated Fatty Acids, Mercury, and Risk of Sudden Cardiac Death in Men: A Prospective Population-Based Study

**DOI:** 10.1371/journal.pone.0041046

**Published:** 2012-07-16

**Authors:** Jyrki K. Virtanen, Jari A. Laukkanen, Jaakko Mursu, Sari Voutilainen, Tomi-Pekka Tuomainen

**Affiliations:** University of Eastern Finland, Institute of Public Health and Clinical Nutrition, Kuopio, Finland; University of Massachusetts Medical School, United States of America

## Abstract

**Objectives:**

Fish consumption has been associated with reduced risk of cardiovascular diseases (CVD), especially sudden cardiac death (SCD). Fish is the major source of long-chain n-3 polyunsaturated fatty acids (PUFA) eicosapentaenoic acid and docosahexaenoic acid. It is also a major source of methylmercury, which was associated with increased risk of CVD in this study population. Impact of interaction between long-chain n-3 PUFA and methylmercury on the SCD risk is unknown.

**Methods:**

A total of 1857 men from the prospective, population-based Kuopio Ischaemic Heart Disease Risk Factor study, aged 42–60 years and free of CVD at baseline in 1984–1989, were studied. Serum long-chain n-3 PUFA was used as the marker for long-chain n-3 PUFA intake and hair mercury as the marker for mercury exposure.

**Results:**

During the mean follow-up of 20.1 years, 91 SCD events occurred. In the multivariate Cox proportional hazards regression models, serum long-chain n-3 PUFA concentration was not associated with the risk of SCD until hair mercury was accounted for; then the hazard ratio (HR) in the highest vs. lowest tertile was 0.54 [95% confidence interval (CI) 0.32 to 0.91, p for trend  = 0.046]. When the analyses were stratified by hair mercury content, among those with lower hair mercury, each 0.5 percentage unit increase in the serum long-chain n-3 PUFA was associated with HR of 0.77 (95% CI 0.64 to 0.93), whereas no association was seen among those with higher hair mercury (p for interaction  = 0.01). Among the individual long-chain n-3 PUFA, docosahexaenoic acid was most strongly associated with the risk.

**Conclusion:**

High exposure to mercury may reduce the benefits of long-chain n-3 PUFA on SCD.

## Introduction

The benefits of fish consumption have been most strongly observed with reduction in fatal coronary heart disease (CHD) or sudden cardiac death (SCD) [1]. As SCD is often preceded by ventricular arrhythmia [2], this has led to the hypothesis that long-chain n-3 polyunsaturated fatty acids (PUFA) from fish may have an antiarrhythmic effect. This hypothesis is supported by animal and in vitro studies [3]–[5]. In observational studies in humans, consumption of fish or long-chain n-3 PUFA from fish, eicosapentaenoic acid (EPA) and docosahexaenoic acid (DHA), has been associated with markedly reduced risk of SCD in participants without history of CHD [6]–[9] and in a general population [10], although not all studies have found an association [11]. In two study populations, blood long-chain n-3 PUFA showed even stronger associations with SCD risk [6], [12]. Trials with fish or fish oil supplements in post-myocardial infarction (MI) patients have also found beneficial effect on the incidence of SCD or fatal CHD [13], [14]. Benefits have not been observed in all trials, however [15], [16].

Fish is the major dietary source of EPA and DHA, but also a major source of methylmercury. We have previously observed an association between mercury exposure and increased risk of cardiovascular disease (CVD) in our Kuopio Ischaemic Heart Disease Risk Factor (KIHD) Study cohort [17], [18]. Mercury exposure also attenuated the inverse association between serum long-chain n-3 PUFA and cardiovascular events, so that the association was observed only in those with low exposure to mercury [18]. However, besides KIHD, the adverse effect of mercury on cardiovascular events has only been found in the retrospective EURAMIC study from eight European countries and Israel [19]. No adverse effects were observed in the Swedish nested case-control studies [11], [20] or in the large nested case-control study in the USA [21].

Although we have previously assessed CVD death as the study endpoint [16], [17], CVD forms of a fairly heterogeneous group of causes of death, most notably CHD. SCD is another clinically important entity within CVD death and as such has gained increasing recent interest, clearly warranting further attention. Furthermore, long-chain n-3 PUFA may have potential antiarrhythmic effects and SCD is often the result of ventricular arrhythmias, unlike other CHD death outcomes or aortic aneurysm rupture.

Because the methods for estimating dietary intakes are imperfect, studies based on estimates of fish or long-chain n-3 PUFA intakes are subject to bias by misclassification, which may have attenuated the observed associations with the risk of SCD in other studies. Only three studies have used blood levels of long-chain n-3 PUFA as exposure [6], [11], [12], and two of those found stronger associations with SCD risk with blood levels than with dietary intakes of long-chain n-3 PUFA [6], [7]. Thus, the aim of our study was to investigate the association between serum long-chain n-3 PUFA, an objective marker of exposure, and risk of SCD in middle-aged or older men without prior CHD and to assess the risk modification by mercury.

## Methods

### Study population

The KIHD study was designed to investigate risk factors for CVD, atherosclerosis, and related outcomes in a population-based, randomly selected sample of men from eastern Finland [22]. The baseline examinations were carried out in 1984–1989. The study sample was composed of 2682 (82.9% of the eligible) men aged 42, 48, 54, or 60 years at baseline. The baseline characteristics of the entire study population have been described [22]. The KIHD study protocol was approved by the Research Ethics Committee of the University of Kuopio and complies with the Declaration of Helsinki. All subjects gave written informed consent for participation.

Subjects with history of CHD at baseline (n = 677) or with missing data on serum long-chain n-3 PUFA (n = 134) or hair mercury (n = 14) were excluded, leaving 1857 men.

### Measurements

Hair and venous blood samples were collected between 8AM and 10AM at the baseline examinations. Subjects were instructed to abstain from ingesting alcohol for three days and from smoking and eating for 12 hours prior to giving the sample. Detailed descriptions of the determination of serum lipids and lipoproteins [23], assessment of medical history and medications [23], family history of diseases [23], smoking [23], alcohol consumption [23], blood pressure [23], and physical activity [24] have been published. Serum C-reactive protein was measured with an immunometric assay (Immulite High Sensitivity CRP Assay, DPC, Los Angeles, CA, USA). Education was assessed in years by using self-administered questionnaire. Annual income was obtained from a self-administered questionnaire. Dietary intake of foods and nutrients was assessed at the time of blood sampling using 4-day food recording [25]. Mercury in hair was determined by flow injection analysis-cold vapor atomic absorption spectrometry and amalgamation [17]. To study the tracking of hair mercury values over time, repeat hair samples were collected and the mercury contents were measured for 21 subjects 4 to 9 years (mean, 6 years) after the baseline examination. Pearson correlation coefficient between the original and the repeat measurements was 0.91.

### Serum fatty acids

Serum esterified and nonesterified fatty acids were determined in one gas chromatographic run without preseparation [26]. Serum fatty acids were extracted with chloroform-methanol. Chloroform phase was evaporated and treated with sodium methoxide, which methylated esterified fatty acids. Quantification was carried out with reference standards purchased from Nu-Check Prep Inc. (MN, USA). Each analyte had individual reference standard and recovery of analytes was confirmed with an internal standard eicosan (arachidic acid C_20_H_40_O_2_). Fatty acids were chromatographed in an NB-351 capillary column (HNU-Nordion, Helsinki, Finland) by a Hewlett-Packard 5890 Series II gas chromatograph (Hewlett-Packard Company, Avondale, Pa, USA, since 1999 Agilent Technologies Inc., USA) with a flame ionization detector. Results were obtained in µmol/L. The coefficient of variation (CV) for repeated measurements of major esterified fatty acids was ∼5%. Because the relative degree of saturation of fatty acids varies among esterified fatty acid types, the esterified fatty acid concentrations were adjusted for serum LDL and HDL cholesterol and triglyceride concentrations. The CV for major nonesterified fatty acids was ∼15%. No adjustment was conducted for nonesterified fatty acids. Total long-chain n-3 PUFA is the sum of sum of EPA (20∶5n-3), docosapentaenoic acid (DPA, 22∶5n-3) and DHA (22∶6n-3).

### Ascertainment of follow-up events

All deaths that occurred by the end of 2009 were checked from the hospital documents, health center wards, and death certificates. The sources of information were interviews, hospital documents, death certificates, autopsy reports, and medico-legal reports [27]. There were no losses to follow-up. The diagnostic classification of events was based on symptoms, electrocardiographic (ECG) findings, cardiac enzyme elevations, autopsy findings (80%), and history of CHD together with the clinical and ECG findings of the paramedic staff. All the documents related to the death were cross-checked in detail by two physicians. Deaths were coded using to the ICD-9th Revision, codes 410 to 414 for non-SCD and 798.1 for SCD; or the ICD-10th Revision, codes I20 to I25 for non-SCD and I46 for SCD.

A death was determined SCD when it occurred either within 1 h after the onset of an abrupt change in symptoms or within 24 h after onset of symptoms when autopsy data did not reveal a noncardiac cause of sudden death. The deaths due to aortic aneurysm rupture, cardiac rupture or tamponade, and pulmonary embolism were not included as SCD.

### Statistical analysis

Cox proportional hazards regression models were used to estimate hazard ratio (HR) in thirds of the predictors. The multivariable-adjusted model (Model 2) included age, examination year, BMI, pack-years of smoking and alcohol intake. Model 3 included the Model 2 and either hair mercury content in the analyses with the long-chain n-3 PUFA or long-chain n-3 PUFA in the analyses with hair mercury. Diabetes; hypertension; income; education years; systolic or diastolic blood pressure; serum LDL and HDL cholesterol and triglycerides, CRP, selenium, alpha-linolenic acid or linoleic acid; blood glucose; family history of CHD; physical activity; living in a rural area; aspirin use; intakes of energy, milk and milk products, meat and meat products, and fruits, berries and vegetables were tested for entry, but did not change the associations (HR change <5%) and thus were not included in the models. Cohort mean was used to replace missing values (<2.1%) in covariates. Statistical significance of the interactions on a multiplicative scale was assessed by likelihood ratio tests using a cross-product term. Tests of linear trend were conducted by assigning the median values for each category of exposure variable and treating those as a single continuous variable. In the analyses with continuous exposure variables, the HRs are shown for each 0.5 unit change, because the average values for the serum long-chain n-3 PUFAs and hair mercury are small. For the tests of quadratic (non-linear) trend, the linear trend variable was squared after centering it at median hair mercury value. Correlations were estimated by Pearson's correlation coefficients. All p-values were 2-tailed (α = 0.05). Data were analyzed using SPSS 19.0 for Windows (SPSS Inc., Chicago, IL, USA).

## Results

### Baseline characteristics

At baseline, the mean ± SD age of the cohort was 52.8±5.3 years. The mean ± SD concentrations were 4.67±1.59% for EPA+DPA+DHA, 1.66±0.91% for EPA, 0.55±0.10% for DPA and 2.46±0.73% for DHA, of all serum fatty acids. The mean ± SD hair mercury concentration was 1.91±1.96 µg/g. Serum EPA+DPA+DHA correlated modestly with hair mercury (r = 0.29, p<0.001). Among the long-chain n-3 PUFA, only DPA concentration was statistically significantly lower in those who experienced a SCD during the follow up, when compared with those who did not (Table 1). In contrast, the mean hair mercury concentrations were significantly higher in those who experienced a SCD (Table 1). There was no difference in fish consumption between those who died of SCD (47.9 g/day, SD 63.7) and the others (45.4 g/day, SD 53.3), p = 0.66.

**Table 1:** Mean baseline concentrations of serum total long-chain n-3 polyunsaturated fatty acids and hair mercury in those who experienced a sudden cardiac death during the follow-up and in those who did not.

	*Participants with sudden cardiac death during follow-up (n = 91)*	*Participants free of sudden cardiac death during follow-up (n = 1766)*	*p-value*
EPA+DPA+DHA, %	4.64 (1.67)	4.68 (1.58)	0.82
EPA, %	1.75 (1.01)	1.66 (0.90)	0.36
DPA, %	0.53 (0.11)	0.55 (0.10)	0.01
DHA, %	2.36 (0.68)	2.46 (0.73)	0.20
Hair mercury, µg/g	2.85 (2.95)	1.86 (1.88)	<0.001

Values are means (SD).

EPA  =  eicosapentaenoic acid, DPA  =  docosapentaenoic acid, DHA  =  docosahexaenoic acid.

At baseline, compared with men with lower serum EPA+DPA+DHA, men with higher concentrations were more likely to be older and have a higher hair mercury concentration, BMI, income and education and higher intake of alcohol and fruits, berries and vegetables (Table 2). They also had more CHD in family. In contrast, men with higher hair mercury concentrations were more likely to smoke, live in a rural area, have less physical activity in leisure-time, lower education and income, and lower intake of fruits, berries and vegetables (Table 3).

**Table 2:** Baseline characteristics according to serum EPA+DPA+DHA.

	*Serum EPA+DPA+DHA tertile (% of all serum fatty acids)*			
	*1 (1.70–3.85%)*	*2 (3.86–4.95%)*	*3 (4.96–15.59%)*	*p for trend*
Number of subjects	619	619	619	
Age, y	52.1 (5.5)	52.5 (5.4)	52.7 (5.2)	0.03
Smoking, %	32	30	28	0.11
Body mass index, kg/m^2^	26.5 (3.4)	26.6 (3.3)	27.1 (3.6)	0.003
Leisure-time physical activity, kcal/day	133 (166)	131 (150)	150 (190)	0.06
Income, euros	13360 (7760)	13390 (9410)	15370 (10670)	<0.001
Education, y	8.8 (3.3)	8.8 (3.5)	9.3 (3.9)	0.01
Hair mercury, µg/g	1.24 (1.41)	1.91 (1.89)	2.59 (2.24)	<0.001
Fish intake, g/d	21 (32)	42 (44)	73 (66)	<0.001
Intake of fruits, berries & vegetables, g/d	233 (151)	233 (157)	267 (182)	<0.001
Alcohol intake, g/wk	56 (90)	80 (130)	86 (125)	<0.001
Living in a rural area, %	30.2	26.7	26.5	0.17
Coronary heart disease in family, %	43.3	44.4	49.4	0.03
Hypertension, %	42.8	40.7	42.0	0.77

Values are means (SD) or percentages.

EPA  =  eicosapentaenoic acid, DPA  =  docosapentaenoic acid, DHA  =  docosahexaenoic acid.

**Table 3:** Baseline characteristics according to hair mercury concentration.

	*Hair mercury tertile*			
	*1 (0–0.83 µg/g)*	*2 (0.84–1.99 µg/g)*	*3 (2.00–15.67 µg/g)*	*p for trend*
Number of subjects	615	623	619	
Age, y	51.2 (5.8)	52.3 (5.3)	53.7 (4.6)	<0.001
Smoking, %	28.5	26.6	34.6	0.01
Body mass index, kg/m^2^	26.4 (3.2)	26.8 (3.4)	27.1 (3.7)	<0.001
Leisure-time physical activity, kcal/day	152 (200)	137 (150)	125 (153)	0.01
Income, euros	15370 (9770)	14900 (9670)	12430 (8380)	<0.001
Education, y	9.8 (3.7)	9.1 (3.8)	8.0 (3.1)	<0.001
Serum EPA+DPA+DHA, %	4.08 (1.20)	4.67 (1.40)	5.27 (1.86)	<0.001
Fish intake, g/d	30 (38)	43 (47)	64 (67)	<0.001
Intake of fruits, berries & vegetables, g/d	257 (163)	245 (162)	231 (168)	<0.001
Alcohol intake, g/wk	65 (115)	78 (124)	78 (112)	0.09
Living in a rural area, %	21.8	25.8	35.7	<0.001
Coronary heart disease in family, %	43.9	47.5	45.7	0.72
Hypertension, %	40.3	41.3	43.9	0.18

Values are means (SD) or percentages.

EPA  =  eicosapentaenoic acid, DPA  =  docosapentaenoic acid, DHA  =  docosahexaenoic acid.

### Serum long-chain n-3 PUFA and risk of SCD

During the mean ± SD follow-up of 20.1±5.6 years (37,326 person-years), 91 SCD events occurred. We did not find statistically significant associations between serum EPA+DPA+DHA in tertiles and risk of SCD after adjustment for age and examination year (Model 1 in Table 4) or with further adjustments for BMI, alcohol intake and pack-years of smoking (Model 2). However, after further adjustment for hair mercury (Model 3), men in the highest vs. the lowest serum EPA+DPA+DHA tertile had a HR of 0.54 (95% CI 0.32 to 0.91). Although the p-value for trend was statistically significant, the risk was reduced already in the second tertile and no further reduction was observed in the highest tertile.

**Table 4:** Hazard ratio of sudden cardiac death in tertiles of serum total long-chain n-3 polyunsaturated fatty acids.

	*Serum fatty acid tertile*				
	*1 (n = 619)*	*2 (n = 619)*	*3 (n = 619)*	*p for trend*	*HR for each 0.5 percentage unit increase*
EPA+DPA+DHA, %	(1.70–3.85)	(3.86–4.95)	(4.96–15.59)		
N of cases (%)	36 (5.8)	22 (3.6)	33 (5.3)		
Model 1	1.0 (Ref)	0.57 (0.33 to 0.96)	0.85 (0.53 to 1.36)	0.64	0.98 (0.92 to 1.05)
Model 2	1.0 (Ref)	0.53 (0.31 to 0.90)	0.70 (0.43 to 1.15)	0.26	0.97 (0.90 to 1.04)
Model 3	1.0 (Ref)	0.47 (0.27 to 0.81)	0.54 (0.32 to 0.91)	0.046	0.94 (0.87 to 1.01)

Values are hazard ratios (95% confidence interval).

Model 1: adjusted for age and examination year.

Model 2: adjusted for Model 1 and body mass index, pack-years of smoking and alcohol intake.

Model 3: adjusted for Model 2 and hair mercury content.

EPA  =  eicosapentaenoic acid, DPA  =  docosapentaenoic acid, DHA  =  docosahexaenoic acid.

Among the long-chain n-3 PUFA, DHA was most strongly and consistently associated with the risk (Table 5). After multivariate plus hair mercury adjustment (Model 3), we found a HR of 0.52 (95% CI 0.30 to 0.90; p for trend  = 0.02) in the highest vs. the lowest tertile. When evaluated continuously, each 0.5%-unit increase in the serum DHA concentration was associated with a 19% (HR 0.81; 95% CI 0.68 to 0.96) lower risk of SCD (Table 5). Further adjustment for EPA and DPA did not appreciably change the associations (data not shown).

**Table 5:** Hazard ratio of sudden cardiac death in tertiles of serum individual long-chain n-3 polyunsaturated fatty acids.

	*Serum fatty acid tertile*				
	*1 (n = 619)*	*2 (n = 619)*	*3 (n = 619)*	*p for trend*	*HR for each 0.5 percentage unit increase*
EPA, %	(0.23–1.21)	(1.22–1.77)	(1.78–8.67)		
N of cases (%)	31 (5.0)	23 (3.7)	37 (6.0)		
Model 1	1.0 (Ref)	0.65 (0.38 to 1.13)	1.03 (0.63 to 1.67)	0.65	1.02 (0.92 to 1.13)
Model 2	1.0 (Ref)	0.63 (0.37 to 1.09)	0.83 (0.50 to 1.37)	0.68	0.98 (0.88 to 1.10)
Model 3	1.0 (Ref)	0.61 (0.35 to 1.05)	0.69 (0.41 to 1.16)	0.26	0.95 (0.84 to 1.07)
DPA, %	(0.24–0.50)	(0.51–0.59)	(0.59–1.19)		
N of cases (%)	42 (6.8)	26 (4.2)	23 (3.7)		
Model 1	1.0 (Ref)	0.63 (0.39 to 1.03)	0.56 (0.34 to 0.93)	0.03	0.25 (0.08 to 0.75)
Model 2	1.0 (Ref)	0.76 (0.46 to 1.25)	0.72 (0.43 to 1.22)	0.22	0.44 (0.15 to 1.30)
Model 3	1.0 (Ref)	0.71 (0.43 to 1.17)	0.64 (0.38 to 1.08)	0.10	0.33 (0.11 to 0.98)
DHA, %	(0.91–2.10)	(2.11–2.65)	(2.66–6.58)		
N of cases (%)	35 (5.7)	29 (4.7)	27 (4.4)		
Model 1	1.0 (Ref)	0.85 (0.52 to 1.38)	0.77 (0.47 to 1.28)	0.32	0.90 (0.78 to 1.05)
Model 2	1.0 (Ref)	0.80 (0.47 to 1.28)	0.68 (0.40 to 1.14)	0.15	0.88 (0.75 to 1.03)
Model 3	1.0 (Ref)	0.69 (0.42 to 1.14)	0.52 (0.30 to 0.90)	0.02	0.81 (0.68 to 0.96)

Values are hazard ratios (95% confidence interval).

Model 1: adjusted for age and examination year.

Model 2: adjusted for Model 1 and body mass index, pack-years of smoking and alcohol intake.

Model 3: adjusted for Model 2 and hair mercury content.

EPA  =  eicosapentaenoic acid, DPA  =  docosapentaenoic acid, DHA  =  docosahexaenoic acid.

With EPA and DPA we found a similar, about 40% lower risk in the highest vs. the lowest tertile after adjustment for the Model 3, but the associations were not statistically significant (Table 5). When evaluated continuously, only DPA was associated with lower risk. Each 0.5%-unit increase in the serum DPA was associated with a 67% (HR 0.33; 95% CI 0.11 to 0.98) lower risk of SCD (Model 3).

### Hair mercury and risk of SCD

Hair mercury was not statistically significantly associated with the risk of SCD when evaluated in tertiles (Table 6). When evaluated continuously, each 0.5 µg/g increase in the hair mercury content was associated with 7% (HR 1.07; 95% CI 1.03 to 1.11) increased risk of SCD, after adjustment for the Model 3. Further detailed adjustment for potential confounders presented in Table 3: smoking (never, former, current), education (four categories), leisure-time physical activity (quartiles), income (quartiles), intake of fruits, berries and vegetables (quartiles), and living in a rural area, had only a very small impact on the associations in tertiles (HR in the highest mercury tertile 1.40; 95% CI 0.81 to 2.43; p for trend  = 0.05) and did not change the association when mercury was evaluated continuously (HR 1.07; 95% CI 1.03 to 1.11). Because the association between hair mercury and risk of SCD appeared to be J-shaped, in post-hoc tests we also evaluated the non-linearity of the association; the p-value for quadratic trend was 0.12 (Model 3).

**Table 6:** Hazard ratio of sudden cardiac death in tertiles of hair mercury content.

	*Hair mercury tertile*				
	*1 (0–0.83 µg/g) (n = 615)*	*2 (0.84–1.99 µg/g) (n = 623)*	*3 (2.00–15.67 µg/g) (n = 619)*	*p for trend*	*HR change for each 0.5 µg/g increase*
Median hair mercury, µg/g	0.45	1.27	3.25		
N of cases (%)	24 (3.9)	22 (3.5)	45 (7.3)		
Model 1	1.0 (Ref)	0.79 (0.44 to 1.42)	1.59 (0.95 to 2.63)	0.02	1.07 (1.03 to 1.11)
Model 2	1.0 (Ref)	0.71 (0.39 to 1.27)	1.32 (0.79 to 2.22)	0.07	1.06 (1.02 to 1.10)
Model 3	1.0 (Ref)	0.75 (0.41 to 1.35)	1.48 (0.87 to 2.54)	0.03	1.07 (1.03 to 1.11)

Values are hazard ratios (95% confidence interval).

Model 1: adjusted for age and examination year.

Model 2: adjusted for Model 1 and body mass index, pack-years of smoking and alcohol intake.

Model 3: adjusted for Model 2 and EPA+DPA+DHA content.

High hair mercury content significantly attenuated the beneficial impact of the long-chain n-3 PUFAs (Table 7). Among those with hair mercury content below the median (<1.28 µg/g), each 0.5%-unit increase in the serum EPA+DPA+DHA was associated with multivariate-adjusted 23% (HR 0.77; 95% CI 0.65 to 0.93) lower risk of SCD, whereas no association was seen among those with higher hair mercury content (p for interaction  = 0.01). A similar association was found with the serum EPA, although the interaction was only borderline statistically significant (p = 0.09). With DPA, among the participants with low hair mercury content, each 0.5%-unit increase, which accounts for nearly doubling the mean serum DPA concentrations, was associated with 86% (HR 0.14; 95% CI 0.02 to 0.94) lower risk of SCD. No statistically significant association was found in those with high hair mercury content (HR 0.70; 95% CI 0.19 to 2.61, p for interaction  = 0.07). The interaction was the most evident with DHA, with each 0.5%-unit increase being associated with 42% (HR 0.58; 95% CI 0.41 to 0.81) lower risk of SCD among those with low hair mercury, but no association was found among those with high hair mercury content (p for interaction  = 0.004). Further adjustment for smoking, education, leisure-time physical activity, income, intake of fruits, berries and vegetables and living in a rural area did not have significant impact on the interactions (data not shown).

**Table 7:** Hazard ratio for sudden cardiac death associated with each 0.5 percentage unit increase in serum long-chain n-3 polyunsaturated fatty acids, stratified by the median hair mercury content.

	*Hair mercury <1.28 µg/g (n = 928)*	*Hair mercury ≥1.28 µg/g (n = 929)*	*p for interaction*
Number of cases (%)	37 (4.0)	54 (5.8)	
EPA+DPA+DHA, % (SD)	4.26 (1.33)	5.09 (1.71)	
	0.77 (0.64 to 0.93)*	1.02 (0.95 to 1.09)	0.01
EPA, % (SD)	1.43 (0.71)	1.89 (1.02)	
	0.73 (0.52 to 1.01)	1.05 (0.94 to 1.17)	0.09
DPA, % (SD)	0.54 (0.09)	0.57 (0.11)	
	0.14 (0.02 to 0.94)	0.70 (0.19 to 2.61)	0.07
DHA, % (SD)	2.29 (0.66)	2.63 (0.75)	
	0.58 (0.41 to 0.81)	1.01 (0.84 to 1.21)	0.004

* Values are hazard ratios (95% confidence interval).

The model is adjusted for age, examination year, body mass index, pack-years of smoking and alcohol intake (Model 2 in Table 2).

EPA  =  eicosapentaenoic acid, DPA  =  docosapentaenoic acid, DHA  =  docosahexaenoic acid.

## Discussion

In this prospective, population-based cohort of men, higher serum long-chain n-3 PUFA concentration, especially DHA, was associated with a lower risk of SCD. The main finding of the study, however, was that the benefits were reduced by high hair mercury content.

The observed beneficial impact of the long-chain n-3 PUFA on the SCD risk is consistent with previous observational studies, where fish or EPA+DHA consumption was associated with lower SCD risk [6]–[9]. The associations were even stronger when blood long-chain n-3 PUFA were used as the exposure, instead of dietary intakes [6], [12]. In these studies the risk of SCD was reduced by 81–90% [6], [12]. We did not find such remarkable risk reductions in our study, possibly because of the high mercury exposure, which attenuated the associations. The average blood concentrations of the long-chain n-3 PUFA were not markedly different in our study, compared to the two other studies [6], [12].

Furthermore, our finding that DHA had the strongest association with the risk is similar to our previous report, where DHA was most strongly associated with the risk of atrial fibrillation [28]. DHA, but not EPA, has prevented arrhythmias in rats [29], and in humans DHA has been beneficial on heart rate [30], [31] and on heart rate variability [32], [33], an independent predictor of SCD [34]. These findings may be explained by the preferential accumulation of DHA over EPA in myocardial cell membranes [5]. DHA may also better reflect long-term fish intake, because it is the primary fatty acid in cell membranes [35], which serve as a repository and contribute to blood fatty acid levels along with acute intake.

The effect of fish oil supplementation on the SCD incidence has not been studied in primary prevention trials, although a large part of SCDs occurs in those without prior known CHD [2]. In trials with post-MI patients, fish consumption or fish oil supplements have reduced the risk of SCD or fatal CHD [13], [14], although not all trials have found benefits [15], [16]. The increasing use of implantable cardioverter-defibrillators has created a possibility to investigate whether fish oil supplementation specifically has an effect on ventricular arrhythmias. However, the results from the trials have been mixed, although in a meta-analysis a trend toward a protective effect was observed in patients with prior CHD [36].

Little is known about the impact of mercury on the SCD risk, but there is fairly good evidence on the relationship between mercury exposure and decreased heart rate variability [37]. In the KIHD study, we have also previously observed that mercury exposure increased the risk of CVD [17], [18] and also attenuated the beneficial impact of serum long-chain n-3 PUFA [18]. Similar results have been found only in the EURAMIC study, a retrospective case-control study that evaluated the association between toenail mercury levels and risk of first non-fatal MI in 684 cases and 724 controls [19]. In the other prospective cohort studies mercury exposure was not associated with increased CVD risk [11], [20], [21]. The nested case-control study in Northern Sweden included only 78 cases of fatal or non-fatal MI and used erythrocyte mercury, a marker of relatively short-term exposure [20]. In this study and in the recent update with a larger number of cases (n = 431) from the same study population [11], erythrocyte mercury was associated with lower risk of MI. The recent study also evaluated the risk of SCD (81 cases), but found no association with erythrocyte mercury [11]. The largest study on the association between mercury exposure and risk of CVD, the nested case-control study in the Nurses' Health Study and the Health Professionals Follow-up Study (1532 cases of non-fatal MI and 832 cases of fatal CHD), found no association between toenail mercury levels and risk of CVD, either [21].

Several explanations may explain the conflicting results. Mercury levels in the KIHD population are higher than in those studies that did not find an association [11], [20], [21]. For example, in the recent Swedish study, the mean erythrocyte mercury concentration in the male controls was 3.95 µg/L, corresponding to a hair concentration of about 0.6 µg/g [11]. In the U.S. study, the mean toenail mercury concentration was 0.44 µg/g in the male controls, corresponding to about 1.2 µg/g in hair [21]. In our study the mean hair mercury concentration was 1.9 µg/g. Thus, in lower concentrations mercury may only be a marker of the benefits of fish consumption and the adverse effects may not be observed until a certain level of exposure is exceeded. This may also explain the lower risk in the middle hair mercury tertile in our study (Table 6). However, in the US study, there was no increase in the risk of CVD even when extreme deciles were compared [21]. The median toenail mercury in the highest decile was 1.0 µg/g, corresponding to about 2.7 µg/g in hair. Thus, the threshold level of exposure may be population-specific, depending on, for example, intake of dietary antioxidants and selenium, or differences in the genetic-based defenses. In the current study and in the previous studies in the KIHD [17], [18], the risk has increased after the hair mercury content has reached about 2.0 µg/g. Hair mercury concentration may also be a marker of some other factors that would explain the higher risk in those with high mercury concentrations. As seen in the Table 3, high hair mercury concentration was associated with several CVD risk factors. Although only smoking and BMI had an effect on the association between hair mercury and risk of SCD, there were too few cases for detailed subgroup evaluation. We cannot completely exclude the possibility that other factors or residual confounding explain at least partly the higher risk in the highest hair mercury tertile.

Interestingly, besides the small difference in the DPA concentrations, the mean serum long-chain n-3 PUFA concentrations were not statistically significantly lower in the SCD cases, whereas the mean hair mercury content was significantly higher (Table 1). This suggests that the cases consumed fish with higher mercury content or that they had genetical differences in the retention and elimination of mercury [38]. The impact of genetic polymorphisms on the mercury metabolism and further on the CVD risk warrants attention.

Several mechanisms may explain the inverse association between serum long-chain n-3 PUFA and SCD. SCD is often preceded by ventricular arrhythmia [2], and experimental studies in animals have reported potential antiarrhythmic effects of long-chain n-3 PUFA [3]–[5]. For example, they can inhibit the cardiac sodium and L-type calcium channels [3], [5], which reduces myocyte excitability. Other potential mechanisms are the beneficial effects on heart rate [39] and heart rate variability [32], [40].

Besides its negative impact on heart rate variability [37], mercury exposure has also been associated with oxidative stress [41], which in turn has been associated with development of arrhythmias [42]. For example, mercury has a high affinity for sulfhydryl groups, so it can bind to and inactivate antioxidative thiolic compounds such as glutathione [43], a major redox buffer in cells. The oxidation hypothesis is supported by the earlier findings from the KIHD, where high hair mercury content was associated with elevated titers of immune complexes containing oxidized LDL [17].

The potential weakness of the study is the relatively low number of SCD events and that fish intake may have changed during the follow-up. On the other hand, the classification of the SCD events was detailed and serum fatty acids and hair mercury are reliable markers of exposure [44]–[46]. Furthermore, potential misclassification of the participants due to single measurement of serum fatty acids and hair mercury would bias associations towards null, not strengthen them.

In conclusion, the result of this study suggest that the beneficial impact of the long-chain n-3 PUFA on the SCD risk may be reduced by high exposure to mercury, an environmental contaminant in fish. Regular intake of fish with high mercury content should be avoided. These include, for example, large and long-living predatory fish like shark, swordfish and tuna [47].
